# Critical Role for AMPK in Metabolic Disease-Induced Chronic Kidney Disease

**DOI:** 10.3390/ijms21217994

**Published:** 2020-10-27

**Authors:** Florian Juszczak, Nathalie Caron, Anna V. Mathew, Anne-Emilie Declèves

**Affiliations:** 1 Laboratory of Molecular and Metabolic Biochemistry, Faculty of Medicine and Pharmacy, Research Institute for Health Sciences and Technology, University of Mons (UMONS), 7000 Mons, Belgium; anne-emilie.decleves@umons.ac.be; 2 Molecular Physiology Research Unit (URPhyM), Namur Research Institute for Life Sciences (NARILIS), University of Namur (UNamur), 5000 Namur, Belgium; nathalie.caron@unamur.be; 3 Division of Nephrology, Department of Internal medicine, University of Michigan, Ann Arbor, MI 48109, USA; amat@med.umich.edu

**Keywords:** AMPK, chronic kidney disease, obesity, diabetes, autophagy, mitochondrial homeostasis, lipid metabolism, lipotoxicity, proximal tubule

## Abstract

Chronic kidney disease (CKD) is prevalent in 9.1% of the global population and is a significant public health problem associated with increased morbidity and mortality. CKD is associated with highly prevalent physiological and metabolic disturbances such as hypertension, obesity, insulin resistance, cardiovascular disease, and aging, which are also risk factors for CKD pathogenesis and progression. Podocytes and proximal tubular cells of the kidney strongly express AMP-activated protein kinase (AMPK). AMPK plays essential roles in glucose and lipid metabolism, cell survival, growth, and inflammation. Thus, metabolic disease-induced renal diseases like obesity-related and diabetic chronic kidney disease demonstrate dysregulated AMPK in the kidney. Activating AMPK ameliorates the pathological and phenotypical features of both diseases. As a metabolic sensor, AMPK regulates active tubular transport and helps renal cells to survive low energy states. AMPK also exerts a key role in mitochondrial homeostasis and is known to regulate autophagy in mammalian cells. While the nutrient-sensing role of AMPK is critical in determining the fate of renal cells, the role of AMPK in kidney autophagy and mitochondrial quality control leading to pathology in metabolic disease-related CKD is not very clear and needs further investigation. This review highlights the crucial role of AMPK in renal cell dysfunction associated with metabolic diseases and aims to expand therapeutic strategies by understanding the molecular and cellular processes underlying CKD.

## 1. Introduction

Progressive decline in renal function leads to chronic kidney disease (CKD) and, ultimately, end-stage renal disease (ESRD) requiring dialysis or transplantation. As of 2017, 9.1% of the world population has CKD, making CKD a public health problem with an enormous economic impact [1,2]. CKD is associated with metabolic syndrome (MetS), a cluster of metabolic disorders, including hypertension, hyperlipidemia, hyperglycemia, and obesity [3]. MetS contributes to the appearance of albuminuria, the first sign of kidney disease in patients with diabetes [4,5,6]. Moreover, accumulating studies report that obesity is an independent risk factor of CKD [7,8,9]. The growing epidemic of obesity contributes to the increased prevalence of type II diabetes and the related kidney complications making diabetic kidney disease the leading cause of ESRD in the developed world [10].

**Figure 1:**
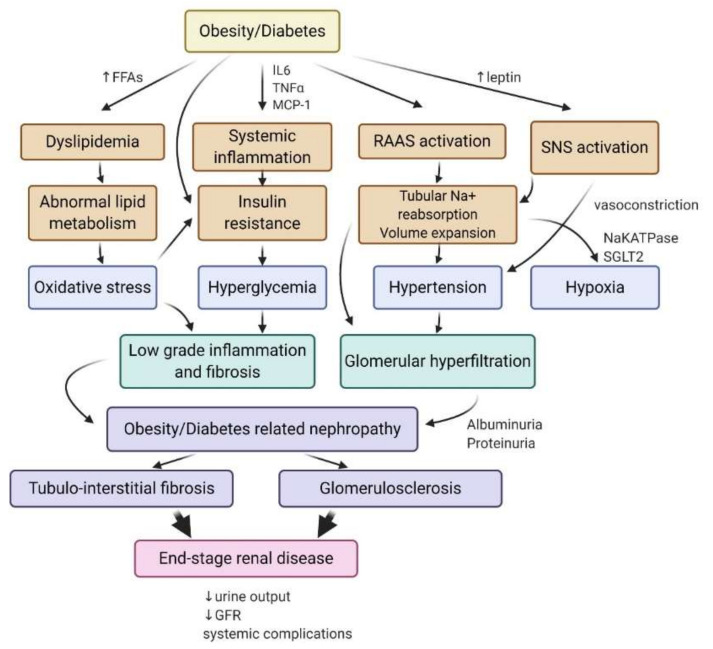
Mechanisms involved in the pathogenesis of diabetes- and obesity-related kidney disease and ultimately end-stage renal disease. Obesity and diabetes initiate systemic disturbances, including systemic inflammation, dyslipidemia, the activation of the SNS, and RAAS that contribute to intrarenal stresses resulting from abnormal lipid metabolism, insulin resistance, and tubular reabsorption of sodium. Hypertension and the associated glomerular hyperfiltration increase albuminuria. Renal oxidative stress and hyperglycemia lead to inflammation and fibrosis that initiate obesity- and diabetes-related nephropathy. Tubulo-interstitial fibrosis and glomerulosclerosis are associated with a progressive decline in the glomerular filtration rate (GFR), loss of nephrons, and ultimately end-stage renal disease. IL6, interleukin 6; TNFα, tumor necrosis factor-α; MCP-1, monocyte chemoattractant protein-1; FFAs, free fatty acids; Na^+^-K^+^ ATPase, sodium–potassium pump; SGLT2, sodium/glucose cotransporter 2; SNS, sympathetic nervous system; GFR, glomerular filtration rate; RAAS, renin–angiotensin–aldosterone system.

Both obesity- and diabetes-related kidney disease are associated with glomerulomegaly, hemodynamic changes, increased albuminuria as well as similar structural and functional changes in the kidney [11,12,13]. Although the lesions found in obesity-induced kidney disease are slightly different from nonobese diabetic patients, it is difficult to discriminate between the effects of obesity vs. the concomitant effects of hyperglycemia and insulin resistance in clinical studies, as there is considerable overlap between diabetic and overweight/obese patients [10]. Adiposity and adipose tissue dysfunction, associated with insulin resistance, lead to the release of proinflammatory cytokines and free fatty acids (FA) in the circulation, as well as changes in the production of adipokines (leptin and adiponectin), which likely contribute to the pathogenesis of obesity and diabetes-induced kidney disease [12,14,15]. Obesity enhances the renin-angiotensin-aldosterone system (RAAS), leptin-induced activation of the sympathetic nervous system (SNS), tubular sodium reabsorption, and volume expansion, leading to hypertension [16]. Increased tubular sodium and glucose reabsorption lead to an enhanced consumption of oxygen in the renal cortex, associated with a lower renal oxygenation due to fluid volume expansion and increased blood flow that contributes to hypoxia induction in renal tissue [17,18]. Obesity-induced hypertension accelerates glomerular hyperfiltration, leading to obesity-related glomerulopathy characterized by glomerulomegaly with focal and segmental glomerulosclerosis lesions [19]. Meanwhile, systemic inflammation and dyslipidemia contribute to the initiation of oxidative stress and insulin resistance, leading to renal low-grade inflammation and fibrosis (Figure 1) [20]. Finally, both obesity- and diabetes-induced kidney disease are characterized by ectopic lipid depositions in the kidney and are associated with direct lipotoxicity in rodents and humans [21,22,23,24,25,26,27]. 

Obesity- and diabetes-related kidney diseases share physiological initiating events and also critical molecular mechanisms of renal cell injury. Studies have demonstrated an essential role of AMP-activated protein kinase (AMPK) dysregulation in obesity- and diabetes-associated kidney disease both in experimental and clinical models [28,29,30,31,32,33]. In several studies, AMPK activators attenuate diabetic nephropathy and improve high fat-induced kidney disease in mice [24,34,35,36]. However, therapeutic approaches to prevent or treat kidney disease in patients with obesity and diabetes are not very specific to these altered pathways. The delay in developing therapeutic strategies to modulate AMPK in obesity- and diabetes-induced kidney disease is partially due to the complexity of the underlying physiological and molecular mechanisms. In this review, we discuss the physiological role of AMPK signaling in renal cells and its dysregulation in obesity- and diabetes-related chronic kidney disease. 

## 2. AMP-Activated Protein Kinase (AMPK): Structure, Renal Expression, and Function

**Figure 2:**
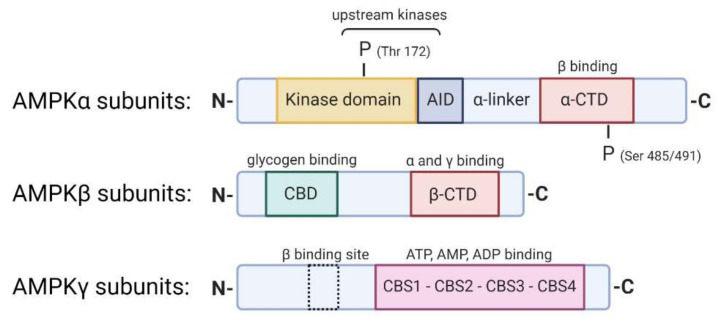
AMPK α, β, and γ subunits. The crystal structure represents the different domains and specific sites of the α, β, and γ subunits that constitute the AMPK heterotrimeric complex. The AMPKα subunits contain a serine/threonine kinase domain at the N-terminus phosphorylated by upstream kinases on the residue Thr172, directly followed by an autoinhibition domain (AID) that maintains the kinase domain inactive in the absence of AMP and a C-terminus domain (α-CTD) that interacts with the β subunits. Phosphorylation of Ser 485/491 residues on the α-CTD negatively regulates AMPK. The AMPKβ subunits have a glycogen-binding domain (CBD) and an α and γ subunit interaction domain (β-CTD). The AMPKγ subunits present four β-synthase (CBS) domains (CBS1-4) that can bind to ATP, AMP, and ADP. AMP binding initiates the allosteric activation of AMPK and promotes phosphorylation of AMPK by upstream kinases.

AMPK is a heterotrimeric complex composed of three different subunits: α, β, and γ (Figure 2). The catalytic subunit α is present in two different isoforms α1 and α2. The β and γ subunits are the regulatory subunits. There are two β-subunits (β1 and β2) and three γ subunits (γ1, γ2, and γ3). Seven different genes encode these multiple protein isoforms: *PRKA1/2*, *PRKAB1/2*, and *PRKAG1/2/3*. The expression of AMPK subunits is tissue-specific and seems to vary in response to stress, suggesting differential functions of each isoform, which are not well understood. The α subunit contains a kinase domain phosphorylated by upstream kinases on Thr172, an autoinhibitory domain (AID), and a β binding domain, essential for the formation of the heterotrimeric complex. AMPKβ contains a carbohydrate-binding molecule (CBM) that allows AMPK to bind to glycogen and an αγ binding domain. The β subunit is implicated in the relocalization of AMPK in glucose-starved states to the lysosome and the mitochondrial membranes with the goal of mitophagy [37]. The structural characteristics of AMPKγ are the four tandem repeats termed cystathionine β-synthase (CBS) motifs that bind adenine nucleotides. The binding of AMP, rather than ADP or ATP, to the AMPKγ subunit leads to the activation of AMPK [38]. 

Renal cells express AMPK extensively. Few studies describe the specific AMPK subunit isoforms expressed in the kidney, as most of the time, the kidney expresses all AMPK isoforms. AMPK is often studied in total kidney lysates, making it difficult to have specific data regarding subunit expression in the whole kidney. However, specific cell populations in the kidney express AMPK isoforms differentially considering their specific functions in metabolism. According to data from The Human Protein Atlas (HPA), AMPK is mostly expressed in cortical tubular epithelial cells, notably on apical surfaces of distal tubules in mice, as demonstrated with immunostaining for Thr172 Phospho-AMPKα [24,39]. The α2 subunit is the predominant catalytic isoform. However, the α1 subunit is also detectable in the tubule but not detectable in the glomeruli. The human kidney expresses β1, β2, and γ2 isoforms. Salatto et al. showed that human and rodent kidneys predominately express AMPK β1 [40]. The α1 isoform is the predominant isoform in the rat kidney, while the kidney expresses both γ1 and γ2 equally [41]. The skeletal muscle expresses the γ3 subunit, but this subunit is not expressed in the kidney. 

### 2.1. An Allosteric Mechanism Activates AMPK

High AMP:ATP ratios under starvation, hypoxia or exercise are associated to the binding of AMP to the regulatory γ subunit, allosterically activating AMPK. The AMP binding favors the phosphorylation of AMPK by upstream kinases on the α subunits at specific Thr172 residues. Furthermore, AMP binding prevents the dephosphorylation of AMPK by protein phosphatases, PP2A, and PP2C. The two central upstream kinases that phosphorylate AMPK are the serine-threonine liver kinase B1 (LKB1) and the calcium/calmodulin kinase kinase β (CAMKKβ). LKB1 activates AMPK in response to low energy states (e.g., exercise, starvation), while CAMKKβ is sensitive to increases in intracellular Ca^2+^ [38]. The transforming growth factor (TGF)-β-activated kinase-1 (TAK1) is also known to phosphorylate AMPK on Thr172 of the α subunit [42]. Other phosphorylation sites, particularly on Ser485/491 (equivalent rodent sequence is Ser487/491), are targeted by other kinases such as Akt through insulin-related signaling or PKA through c-AMP-related signaling pathways. However, these phosphorylations lead to the negative regulation of AMPK by blocking its phosphorylation at Thr172 by upstream kinases [43,44,45]. 

### 2.2. AMPK Exhibits a Dual Function in Cell Metabolism

First, AMPK decreases ATP consumption by inhibiting anabolic pathways, including lipid, glycogen, and protein synthesis. Secondly, AMPK activates catabolic pathways by increasing lipid oxidation, glucose uptake, autophagy flux, and mitochondrial biogenesis. The following sections will discuss these pathways in the context of obesity- and diabetes-related kidney disease. Energy sensing by AMPK is particularly relevant in renal cells because these cells are strongly dependent on the regulation of energy metabolism for tubular transport. 

AMPK regulates the Na^+^-K^+^-ATPase (NKA), epithelial sodium channel (ENaC), the Na^+^-K^+^-2Cl^−^ cotransporter (NKCC), Cystic fibrosis transmembrane conductance regulator (CFTR), and other ion transport proteins in the kidney [41]. AMPK plays an essential role in kidney homeostasis, as evidenced by AMPK knockdown studies. Kidney-specific deletion of both AMPKα subunits displayed salt and water wasting defects [46]. Both AMPKα2 knockout mice and endothelium-specific knockdown of AMPKα2 display an increased angiotensin-converting enzyme (ACE)-levels [47]. Specific renal physiological processes, such as ion transport, blood pressure control, and nitric oxide production, involve AMPK [48]. Tubular epithelial-specific deletion of LKB1, the primary upstream kinase of AMPK, is associated with de-differentiation of tubule epithelial cells, fibrosis, and inflammation, mediated by AMPK and its downstream metabolic effects [33]. On the other hand, uncontrolled sustained activation of AMPK in a Wolff−Parkinson−White Syndrome model led to a disastrous accumulation of glycogen in the kidney and subsequent impairment of renal function [49]. While these genetic studies of AMPK highlight the importance of a tight regulation of AMPK activity in the kidney during physiological conditions, AMPK impairment in the kidney’s response to metabolic stress initiates deleterious outcomes.

## 3. AMPK Activity in Obesity and Diabetes-Induced Chronic Kidney Disease (CKD)

Type II diabetes, obesity, and MetS are characterized by lipid accumulation and hyperglycemia, which is perceived by the cells as a nutrient excess. According to the classic view, the cells respond to this high-energy state by a decreased AMPK activity. However, many cellular and molecular events directly or indirectly linked to metabolic disturbances in the whole body inhibit AMPK [50]. As previously mentioned, AMP: ATP ratio during physiological conditions or adaptations such as exercise or starvation strongly correlate with AMPK activity. In obesity and diabetes, additional mechanisms, independent of the AMP: ATP ratio, might also promote the reduced AMPK activity. With nutrient excess, the energy state of the cell favors an altered redox status with higher NADH production through glycolysis [51]. This increase in NADH, in turn, leads to reduced activity of Sirtuin 1 (SIRT1), an NAD^+^-dependent deacetylase, that is a potent activator of LKB1, therefore favoring the dephosphorylated state of AMPK [52,53,54]. Recently, Kikuchi et al. further demonstrated in CKD-induced by a subtotal nephrectomy in mice that AMPK activity was decreased in renal tissue in spite of high energy demand. CKD-associated acidosis and uremic metabolites were particularly implicated in the sensing failure to upregulate AMPK despite an increased AMP to ATP ratio [55].

Elevated circulating hormones such as insulin and leptin in metabolic diseases downregulate AMPK by inducing inhibitory phosphorylation on different serine residues. For example, insulin decreases AMPK activity through phosphorylation on Ser485 and Ser491 of AMPKα subunits by the Akt pathway. At the same time, leptin induces AMPK inhibition by p70S6 kinase downstream of Akt [56,57]. Additionally, low adiponectin levels in obesity and type II diabetes could also decrease AMPK activation via its receptor, AdipoR1. Adiponectin knockout mice have decreased AMPK activity, while AMPK activity correlates with adiponectin levels in an obesity model [58]. In high-fat and high-sucrose diet models, tissue expression of adiponectin receptors was dysregulated, highlighting a mechanism of adiponectin resistance in peripheral tissues that could also contribute to impaired AMPK activity [59,60]. More recently, the treatment of *db*/*db* mice with an activator of adiponectin, AdipoRon, showed the upregulation of phosphorylated AMPK in the kidney along with reduced inflammation and lipotoxicity [30]. Finally, obesity and diabetes are associated with inflammation and oxidative stress that are both recognized to inhibit AMPK [61]. The pro-inflammatory cytokine TNFα is known to suppress AMPK activity via the induction of protein phosphatase 2C (PP2C) in skeletal muscle in vitro and in vivo, suppressing fatty acid (FA) oxidation and promoting insulin resistance [62].

## 4. AMPK in Renal Transport

The role of AMPK in ion transport has been described in earlier reviews [63,64]. However, very little is known about the role of AMPK in renal transport in the setting of obesity and diabetes. Both obesity and insulin resistance are associated with significant changes in tubular transport, leading to electrolyte disorders such as hypomagnesemia, hyper/hyponatremia, hyper/hypokalemia, and hyper/hypocalciuria [65,66]. Interestingly, AMPK interferes with Na^+^-handling, notably by activating the primary transport, the NKA [67]. Xiao et al. demonstrated that the AMPK pathway is the principal regulator of NKA signaling as the hyperuricemia-induced renal tubular injury impairs NKA. Moreover, AMPK activation exerts protective effects regarding NKA-mediated mechanisms of tubular injury by regulating NKA expression and reducing lysosomal NKA degradation [68]. However, AMPK activation also downregulates the ENaC expression in the distal tubule [69,70] while it seems to enhance the stimulation of the NKCC that is responsible for sodium reabsorption in the thick ascending limb [46,71]. Nevertheless, the use of knockout mice for AMPK subunits demonstrated only a moderate role for AMPK in renal sodium handling, suggesting that AMPK might only play a secondary modulatory role [72]. Furthermore, AMPK downregulates many other renal transporters such as Na^+^-coupled phosphate transporter (NaPi-IIa) or CFTR [73,74]. Therefore, though reduced renal AMPK activity in metabolic diseases may contribute to salt and water imbalance in obesity- or diabetes-related disease, the role of AMPK in renal tubular handling in the metabolic disease associated kidney disease needs further investigation. 

## 5. AMPK and Renal Lipid Metabolism

**Figure 3:**
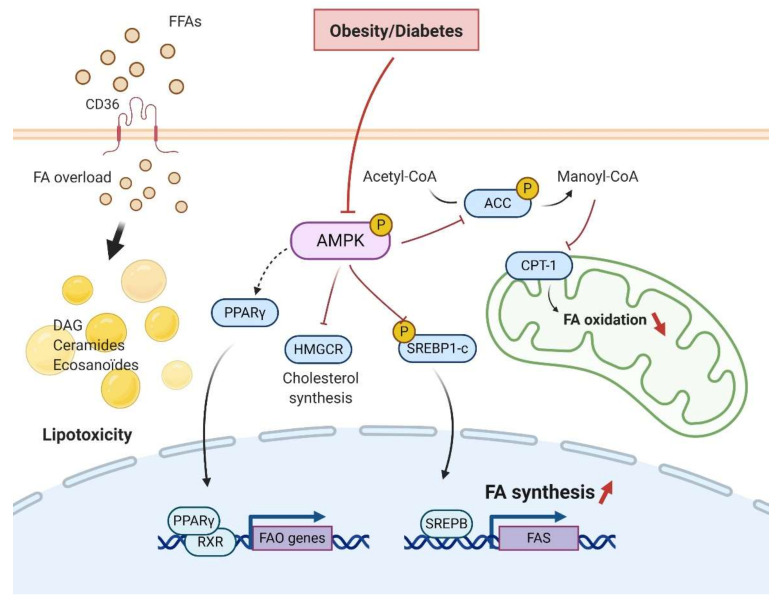
Impaired regulation of lipid metabolism by AMPK in renal cells in obesity/diabetes. Inhibition of AMPK and the increased FA overload lead to a decreased mitochondrial FA oxidation and an enhanced lipogenesis, initiating lipotoxicity in renal cells. Inhibitory phosphorylation of ACC by AMPK is removed, allowing for malonyl-CoA production, leading to inhibition of CPT-1 activity and suppression of FA oxidation in mitochondria. HMGCR and SREBP1-c are enhanced and initiate cholesterol and FA synthesis that contributes to a general increase of lipogenesis, leading to further lipid accumulation in renal cells. Excess FA content associated with impaired AMPK pathway and related lipid metabolism initiates ectopic lipid accumulation in renal cells, including harmful lipid metabolites (DAG, ceramides, and eicosanoids) that cause lipotoxicity. AMPK, AMP-activated protein kinase; ACC, Acetyl-CoA carboxylase; CPT1, Carnitine palmitoyltransferase I; PPARγ, peroxisome proliferator-activated receptor γ; HMGCR, 3-hydroxy-3-methyl-glutaryl-coenzyme A reductase; RXR, retinoid X receptor; SREBP, sterol regulatory element-binding proteins; FAS, fatty acid synthesis; FAO, fatty acid oxidation; DAG diacylglycerol; FFAs, free fatty acids. The arrow indicates whether the regulation is activating (black) or inhibitory (red). The dashed arrow indicates a potential regulation.

Diabetic nephropathy and obesity-induced CKD are both linked to renal lipid accumulation and abnormal lipid metabolism (Figure 3) [24,75,76]. Activated AMPK induces the inhibitory phosphorylation of Acetyl-CoA Carboxylase (ACC), the rate-limiting step of FA synthesis, reducing the production of malonyl-CoA. Decreased malonyl-CoA promotes the activation of carnitine palmitoyltransferase-1 (CPT-1), allowing the entry of FA into the mitochondria for β-oxidation. Therefore, a direct consequence of AMPK dysfunction in the kidney is the increase of ACC activity, likely contributing to lipogenesis and ultimately lipid accumulation. Mice fed a high-fat diet confirm dysregulated AMPK and the consequent renal accumulation of cholesterol and triglycerides (TG) [24,77]. 

Obesity and diabetes increase the expression of Sterol Regulatory Element-binding Proteins (SREBP) that are implicated in fatty acid and cholesterol metabolism [78,79]. SREBP-1c knockout mice fed a high fat diet (HFD) do not accumulate renal lipids [80]. However, transgenic mice overexpressing SREBP-1a demonstrated elevated renal TG content with glomerulosclerosis and proteinuria [78]. Though this study did not probe AMPK activity, AMPK is a direct upstream kinase of SREBP in the liver [81,82]. Activation of AMPK resulted in inhibition of SREBP1-c and attenuation of lipogenesis in hepatocytes [83]. Indeed, Li et al. demonstrated that SREBP1-c phosphorylation at Ser372 by AMPK in the liver led to the inhibition of the transcriptional activity of SREBP1-c by decreasing its cleavage and nuclear translocation [83]. SREBP family of proteins induces FAS by inducing transcriptional expression of FAS genes [84]. Particularly, SREBP1-c contributes to the increased de novo synthesis of fatty acids by regulating both FAS and ACC expression [85]. Moreover, calcium channel blocker Nifedipine decreased AMPK activity and increased SREBP1/2 and intrarenal lipids [86]. These studies confirm the role of AMPK dysregulation in renal lipid accumulation via the SREBP pathway. 

Kume et al. demonstrated an increase in renal TG content and marked neutral lipid accumulations in both the glomeruli and tubular compartment with overexpression of the peroxisome proliferator-activated receptor gamma (PPARγ), while heterozygous PPARγ mice were protected [76]. This study demonstrated decreased AMPK activity, but no link between AMPK and PPARγ was discussed. Interestingly, several independent studies have highlighted a cross-regulation of AMPK and PPARγ, suggesting an expanded regulation of lipid metabolism by AMPK [87,88,89]. 

On the other hand, HFD fed mice demonstrated decreased lipolysis and AMPK activity [24]. Cholesteryl esters and phosphatidyl contents but not TG were increased along with a significant increase of lipid droplets in proximal tubular cells (PTC). AICAR, a specific AMPK activator, prevented all these changes. In that study, AMPK activation in HFD mice also prevented cholesterol synthesis by the regulation of the rate-limiting cholesterol synthesis enzyme 3-hydroxy-3-methylglutaryl-CoA reductase (HMGCR) [24]. Other groups also confirmed lipid droplet accumulation in PTC [21,22,23,24,25,26,27]. Under physiological conditions, the primary role of PTC is the active reabsorption of filtered sodium. This process requires a large amount of ATP, mostly provided through the mitochondrial β-oxidation of FA. Inhibition of FA oxidation induced increased intracellular lipid depositions, cellular de-differentiation, and cell death in the kidney [90,91]. In PTC, FA can also be taken up via the fatty acid translocase (CD36) and the fatty acid-binding proteins (FABP). FA can then be used to supply energy. CD36 was upregulated in kidney biopsies of diabetic patients [92]. Moreover, CD36 upregulation in the kidney led to the inhibition of AMPK activity and subsequent lipotoxicity [86]. 

Excess intracellular FA content can lead to the accumulation of toxic lipid metabolites such as diacylglycerol (DAG) and ceramides. Both the DAG and ceramide are known to be involved in insulin resistance and inflammation. Both are endogenous activators of protein kinase C (PKC) and PP2A [93]. Moreover, the downregulation of AMPK activity through the PKC-dependent Ser487 phosphorylation has been demonstrated in human endothelial cells [94]. Using lipidomics, Declèves et al. demonstrated HFD-induced dysregulation of lipid metabolism and particularly highlighted eicosanoids, which are implicated in the inflammatory response in the kidney. Direct activation of AMPK with AICAR reduced arachidonic acid and docosahexaenoic acid-derived metabolites in the kidney, thus improving the eicosanoid pathway and associated lipotoxicity [95]. Therefore, the altered lipid metabolism in the kidney, characterized by an imbalance between FA accumulation and degradation seems primarily mediated by AMPK. 

## 6. AMPK and Renal Glucose Metabolism

In conditions with abundant glucose, AMPK promotes *GLUT4* gene expression, glucose uptake, and glycolysis in insulin-sensitive cells. Several studies have reported the role of AMPK in mediating glucose transport in podocytes and insulin resistance in diabetic nephropathy [96,97]. Indeed, podocytes display a complex cellular morphology that requires a sustained energy supply to maintain cytoskeletal remodeling. In podocytes, the mitochondrial abundance is low and anaerobic glycolysis represents the predominant energy source that makes them uniquely sensitive to insulin [98]. Although insulin-stimulated glucose uptake is mostly independent of AMPK, Rogacka et al. demonstrated that AMPK activity is essential for insulin sensitivity in podocytes. Moreover, insulin resistance in podocytes cultured in high glucose medium was closely related to AMPK activity [96]. Using pharmacological and genetic approaches, they suggested that the involvement of AMPK in PTEN (phosphatase and tensin homolog) regulation in cultured podocytes was disturbed in high glucose conditions leading to decreased insulin sensitivity. Insulin mediates AMPK regulation through transient receptor potential canonical channel 6 (TRPC6) activation [99]. TRPC6 is a nonselective Ca^2+^ channel protein that is implicated in the pathophysiology of kidney diseases [100]. In diabetic nephropathy, TRPC6 is upregulated, suggesting a putative role of TRPC6 expression in the progression of diabetic nephropathy pathology. Wang et al. investigated the effect of TRPC6 knockout on diabetic kidney disease in the Akita model of type I diabetes [101]. They surprisingly demonstrated a protective effect of TRPC6 downregulation on proteinuria and albuminuria. This role of TRPC6 is contrary to promoting the progression of glomerular impairment associated with insulin resistance in vivo and in podocyte cultures. 

Like AMPK, Sirtuin 1 (SIRT1) acts as a nutrient sensor in cells. AMPK and SIRT1 are reciprocally regulated and share common targets. The expression of SIRT1 is significantly reduced in animal models of diabetes and in diabetic patients. Moreover, podocyte-specific overexpression of SIRT1 led to improvements in glomerular and tubular injuries in diabetic mice [102]. SIRT1 downregulates AMPK activity in podocyte cultures. SIRT1 suppresses the stimulation of AMPK by insulin, suggesting that the SIRT1/AMPK pathway is essential in podocytes for proper insulin response [103]. Both AMPK and SIRT1 promote autophagy flux in renal tissue, regulate mitochondrial homeostasis, and antioxidative defense. AMPK mediates the hypoglycemic and renoprotective effects of Salidroside (plant glycoside) by downstream activation of SIRT1 [104]. 

In the proximal tubule, glucose reabsorption mostly occurs via the Na^+^-glucose cotransporter SGLT2 and to a lesser extent via SGLT1 in later segments of the proximal tubule. Glucose leaves the basolateral membrane through GLUT1 and GLUT2 [105]. Although the AMPK regulation of SGLT2 has not been clearly addressed, the use of SGLT2 inhibitors mimics the cellular response to starvation and indirectly activates AMPK, leading to the improvement of nephropathy development [106]. However, it has been demonstrated that AMPK activates SGLT1-dependent glucose transport in colorectal cells and cardiomyocytes that needs to be confirmed in the renal tissue [107,108]. Moreover, AMPK activation increased *GLUT1* gene expression in rat kidneys and is associated with enhanced activity of GLUT2 in murine intestinal tissue [64].

## 7. AMPK and Renal Mitochondrial Function and Dysfunction

### 7.1. Mitochondrial Biogenesis and Dynamics

As already mentioned, the kidneys are highly metabolic organs, rich in mitochondria to meet their enormous energy demands. PTCs have a high number of active transporters that require energy to reabsorb ions, glucose, or other nutrients. Maintaining the mitochondrial function is, therefore, essential to sustain the energy demand and kidney function. AMPK activity promotes mitochondrial homeostasis by regulating mitochondrial biogenesis and dynamics and limiting reactive oxygen species (ROS) formation [38,109]. There is a very substantial body of evidence demonstrating the mitochondrial protection exerted by AMPK, notably by activating peroxisome proliferator-activated receptor gamma co-activator 1-alpha (PGC-1α) [110,111,112,113]. PGC-1α is a transcriptional co-activator and the master regulator of mitochondrial biogenesis. Upon activation, it migrates to the nucleus to activate different transcription factors, including nuclear respiratory factors 1 and 2 (NRF-1 and -2). These, in turn, activate the expression of nuclear-coded respiratory chain proteins and the expression of mitochondrial transcription factor A (Tfam), driving replication of mitochondrial DNA and transcription [114]. 

Mitochondrial dysfunction has been well documented in metabolic disease-induced CKD (reviewed in [115,116]) and is a critical player of the pathogenesis [117]. Szeto et al. demonstrated that the use of SS-31, a mitochondrial-targeted antioxidant, preserved mitochondrial structure in HFD mice and led to the improvement of glomerular and tubular injuries. Interestingly, these effects were attributed to restored AMPK activity, suggesting that mitochondria integrity is essential to maintain AMPK phosphorylation [118]. Even in diabetic nephropathy, the restoration of AMPK activation via AICAR restored mitochondrial function and superoxide production, in association with PGC1-α activation, reduced kidney injury. In another study, treatment with Chloroquine in both in vitro and in vivo diabetic environments enhanced AMPK phosphorylation and led to mitochondrial biogenesis and an improved balance of mitochondrial fusion/fission proteins [119]. Human osteosarcoma cells demonstrate the involvement of AMPK in mitochondrial dynamics (fusion/fission/mitophagy) [120]. Toyama et al. found a new AMPK target protein, the mitochondrial fission factor (MFF), which is the primary receptor for the dynamin-like protein Drp1 involved in the mitochondrial fission mechanism. Later, the same AMPK/MFF/Drp1 axis was dysregulated in mesenchymal stromal cells as well. Renal mitochondrial fragmentation has been observed in both in vitro and in vivo models of diabetic nephropathy that was reversed by both AMPK activators, AICAR and Metformin. Interestingly, in that study, the improvement in mitochondrial fragmentation by AMPK activators was correlated with activation of PGC1-α and the consequent restoration of Drp1 and Mfn1 expression in tubular cells [121]. 

### 7.2. AMPK and Oxidative Stress

**Figure 4:**
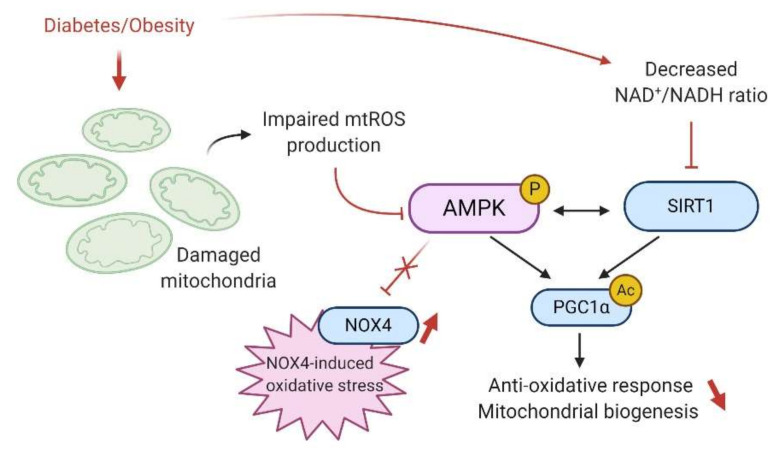
Schematic representation of the proposed mechanism by which diabetes/obesity leads to a decreased antioxidative response mediated by AMPK and enhanced oxidative stress in the renal cell. Regulation of mitochondrial production of reactive oxygen species (ROS) is required for the proper adaptation of the renal cell to metabolic stress. Diabetes and obesity impair renal mitochondrial ROS, which is associated with decreased AMPK. AMPK inhibition and decreased SIRT1 activity lead to a maladaptive antioxidative response through PGC1-α activity suppression, notably decreasing mitochondrial biogenesis. Decreased AMPK also results in increased NOX4-dependent ROS production, further increasing oxidative stress in renal cells. The arrow indicates whether the regulation is activating (black) or inhibitory (red).

Oxidative stress plays a crucial role in the development and the progression of diabetic nephropathy and obesity (Figure 4). Because renal cells are metabolically very active and contain many mitochondria, they are highly vulnerable to ROS generation and therefore to oxidative damage [116]. Under physiological conditions, ROS are generated as standard products of aerobic metabolism, acting as intracellular signaling messengers. Therefore, ROS such as the superoxide radical (O_2_^•−^) and hydroxyl radical (^•^OH) or even the nonradical hydrogen peroxide (H_2_O_2_) are continuously produced during cell metabolism and are eliminated by the antioxidant defenses that include superoxide dismutase (SOD), catalase, peroxidases and the glutathione system to prevent cellular protein damage or lipid peroxidation. However, under nutrient stress such as during chronic hyperglycemia or hyperlipidemia, overproduction of ROS due to the disruption of the redox status occurs, leading to renal inflammation, fibrosis, and impairment of organ structure and function [8,122]. 

In the kidney, the mitochondria are the primary source of ROS production due to a leakage from the mitochondrial electron-transport chain. Regarding AMPK and oxidative stress, it has been well established that AMPK activity plays a crucial role in maintaining redox homeostasis in the cells. AMPK is essential in the suppression of ROS and acts thus as an oxidative stress defense. Different models have described a link between reduced AMPK activity and mitochondrial ROS (mtROS) [112,115,116,117]. Physiologically, mtROS promotes noncanonical activation of AMPK, triggering antioxidative responses through PGC1-α activation and regulating mitochondria homeostasis by activation of the mitophagy process via AMPK-mediated phosphorylation of the Unc-51 like autophagy activating kinase ULK-1 [109]. AMPK regulates gene expression of several antioxidative genes, including *catalase*, *SOD2*, *UCP2*, and *SIRT3* [123]. Notably, AMPKα or PGC1α deficiency increases oxidative stress in cells and cannot overcome ROS-induced damage and mitochondrial homeostasis disruption [109]. 

Under pathological conditions, the contribution of ROS to the development and the progression of kidney disease is controversial. For example, Ruggiero et al. demonstrated that the kidneys of mice fed an HFD could maintain mitochondrial biogenesis and bioenergetics, despite evidence of renal oxidative stress, suggesting an adaptive response to the free FA overload [124]. Still, Dugan et al. reported a reduced superoxide production associated with suppression of mitochondrial activity in the type I diabetic model [29]. The decreased mtROS was concomitant with a decreased AMPK activity and led to further inhibition of mitochondria in a feedback loop manner. Interestingly, AICAR treatment restored AMPK activity and the mitochondrial superoxide production reversing the hallmarks of diabetic kidney disease such as glomerular matrix expansion and albuminuria [29]. Sharma et al. proposed that stimulation of mitochondrial function and superoxide production by AMPK agonists would be associated with better outcomes in the diabetic kidney [125]. However, mtROS production inhibits AMPK activity through the SIRT1 pathway. Indeed, mtROS mediate DNA breaks in the nucleus that require the activation of PARP (repair enzyme, poly adenosine diphosphate ribose polymerase), another NAD^+^-dependent enzyme. Therefore, upon PARP activation, the pool of cellular NAD^+^ is reduced, decreasing NAD^+^ availability for SIRT1, reducing the phosphorylation of AMPK through LKB1 activation [126]. In conclusion, the direct link between AMPK and mtROS production has not been fully understood and will require further investigation in the context of diabetes- and obesity-induced CKD.

NADPH oxidases (NOXs) are another important source of ROS in renal cells. NOX-derived ROS are involved in vascular oxidative stress and endothelial dysfunction in obesity. NOX4 is the most abundant NOX isoform in the kidney and predominantly generates H_2_O_2_ that also plays physiological roles in renal cells [127,128]. Recently, Muñoz et al. demonstrated reduced endothelial NOX4 expression associated with decreased H_2_O_2_ generation and H_2_O_2_-mediated vasodilatation in obese rats [129]. NOX4 is upregulated in both diabetic nephropathy and obesity-related CKD [130,131]. The downstream consequences of NOX4 activation in the kidney lead to increased inflammation and fibrosis that is mostly dependent on AMPK signaling. Indeed, AMPK activation reduced NOX4 and ROS production and subsequently inhibiting NFκB and TGF-β-mediated fibrosis. He et al. demonstrated that in renal fibroblast cells and *db/db* mice, high glucose-induced ROS generation is mainly derived from NOX4 and not NOX1 and NOX2. NOX4 activation led to the proliferation and activation of resident fibroblasts and, ultimately, renal fibrosis. Resveratrol treatment in *db/db* mice prevented NOX4 expression primarily due to increased AMPK activity [132]. Similarly, numerous recent studies highlighted the beneficial effects of antioxidants or other compounds against renal injuries implicating AMPK/NOX4/ROS [133,134,135,136,137,138,139]. Furthermore, two independent studies reported that Sestrin-2 mediated activation of AMPK inhibits ROS production, podocyte impairment in the hyperglycemic condition, and suppresses NOX4 in glomerular mesangial cells [140,141]. p53 and proapoptotic protein PUMA (p53-up-regulated modulator of apoptosis) in diabetic podocytes and glomeruli are promoted by AMPK/NOX4 dysregulation and reversed by AICAR treatment [32]. Therefore, many studies have demonstrated AMPK dependent NOX4 downregulation in the diabetic kidney, suggesting a protective role of NOX4 in diabetic nephropathy.

## 8. AMPK and the Regulation of Renal Autophagy and Mitophagy

Autophagy is a complex and highly regulated process of self-degradation of cellular components. In the kidney, basal autophagy flux is essential to maintain cellular homeostasis and renal function. Kidney-specific autophagy-deficient mice revealed evidence of damaged mitochondria, protein aggregates, endoplasmic reticulum stress, podocyte, and proximal tubular cell loss, and progressive impairment of renal function [142]. The role of AMPK in the regulation of the autophagy flux has been extensively investigated in liver, muscle, brain, or adipocytes, whereas only a few studies explore the role of AMPK in renal autophagy [143,144,145,146]. AMPK positively regulates the autophagy process through phosphorylation of essential target proteins, mainly through the mechanistic target of rapamycin (mTOR) and ULK-1 [147]. Upon activation, AMPK inhibits mTOR and activates ULK-1, which, in turn, activates autophagy [148]. The inhibition of mTOR by AMPK is either by direct phosphorylation or through phosphorylation of the tuberous sclerosis complex 2 (TSC2) protein that then reduces mTOR activity [149]. Interestingly, the high basal level of autophagy observed in podocytes is mainly regulated by the AMPK/ULK-1 axis rather than the mTOR pathway, suggesting that the activation of autophagy by AMPK might be cell-type dependent in kidneys [150]. Moreover, AMPK phosphorylates Raptor leading to a decrease in mTORC1 activity [151]. Finally, AMPK activity leads to an increased intracellular pool of NAD^+^ that allows an increase of SIRT1 activity. SIRT1 targets FOXO1 and FOXO3 in the nucleus and autophagy-related genes *Atg5*, *Atg7*, and *Atg8*, which positively regulate autophagy [152].

Obesity or diabetes can lead to impairment of autophagy flux in renal cells [153,154]. HFD/obesity-induced autophagy impairment has been described in rodent models and obese patients [27,155]. Yamamoto et al. described the stagnation of the autophagy flux as a novel mechanism of renal lipotoxicity in a model of mice fed an HFD. They demonstrated that long term lipid overload was associated with impairments of the lysosomal system and to a consequent accumulation of phospholipids, probably of mitochondrial origin. This lipotoxicity was associated with a sustained mTOR activation, but the association with AMPK was not explored. Excessive fat also induces constitutive activation of mTORC1 that directly inhibits AMPK activity, suggesting a link between mTOR and AMPK [156,157]. However, AMPK-mediated activation of autophagy protects against renal injury, particularly in AKI [158]. Regarding metabolic disease-related CKD, Yamahara et al. demonstrated that obesity significantly suppressed autophagy in PTC of both mice and humans. Notably, they found that AMPK was implicated in FFA-albumin–induced autophagy in PTCs. In PTCs, AMPK downregulation was associated with decreased autophagy induction by albumin-binding FFA, suggesting that AMPK may be essential for maintaining proper autophagy flux. In obesity, AMPK activity remains unaltered by FFA-albumin overload, but its activity failed to decrease the hyperactivation of the mTORC1 signal, which leads to the obesity-mediated exacerbation of proteinuria-induced tubulointerstitial damage. Delayed activation of AMPK with fenofibrate and AICAR was associated with subsequent autophagy activation in the kidney of mice on HFD [24,159]. Antioxidant drugs such as Berberine or Mangiferin improve autophagy flux in diabetic nephropathy through the activation of AMPK [160,161,162].

There is no direct evidence of the role of AMPK in mitophagy pathways. However, activated ULK-1 translocates to damaged mitochondria and phosphorylates FUN14 domain containing 1 (FUNDC1) protein, which is considered a critical player of mitophagy, allowing the formation of an autophagosome around damaged mitochondria [163]. Therefore, AMPK-induced ULK-1 activation may be a crucial mediator of mitophagy [164]. In the study of Yamamoto et al., the induction of the mitophagy pathway protects PTC from mitochondrial dysfunction. Activation of AMPK with AICAR or Metformin led to an increase of autophagy flux and removal of damaged mitochondria in streptozotocin-induced diabetic nephropathy [121]. Finally, AMPK is implicated in lipophagy, the autophagic degradation of lipid droplets (LDs). Lipophagy and lipolysis of LDs for intracellular lipid mobilization are preceded by the targeted autophagy of Perilipin2 (PLIN2). This process is controlled by AMPK-dependent phosphorylation of PLIN2, suggesting a new pathway to regulate lipid metabolism by AMPK through the regulation of lipophagy [165]. The precise role of AMPK in the regulation of autophagy, mitophagy, or lipophagy in renal cells needs further investigation. However, AMPK dysregulation inhibits autophagy and participates in the pathogenesis of obesity and diabetic CKD. AMPK activation may be a valuable way of restoring the autophagy process in metabolic disease-induced CKD.

## 9. AMPK and Sirtuins

The sirtuins are members of the Class III deacetylases. So far, seven forms of sirtuins have been identified (SIRT1-7) [166]. Specifically, SIRT 1 and SIRT3 are induced by caloric restriction and associated with AMPK [167]. The AMPK/SIRT1/PGC1-α pathway acts as a crucial network to maintain mitochondrial homeostasis (already discussed in this review) [168,169]. However, recent studies highlight an AMPK/SIRT3 axis in disease. Deacetylation accounts for a crucial mechanism to regulate the activity of many substrates involved in energy metabolism [170]. SIRT3 is an NAD^+^-dependent deacetylase, mainly localized in mitochondria. SIRT3 plays a significant role in mitochondrial homeostasis by regulating the mitochondrial respiratory chain and ATP production. SIRT3 promotes FA oxidation through the deacetylation of long chain acyl-CoA dehydrogenase (LCAD) [171] and also exerts an antioxidant activity by targeting the superoxide dismutase 2 (SOD2) and the isocitrate dehydrogenase (IDH2) [166,172]. More interestingly, the cytosolic form of SIRT3 activates LKB1, which in turn activates AMPK in primary cardiomyocytes [173]. Therefore, reduction in SIRT3 expression in a setting of obesity could, in part, explain the reduction in AMPK activity. Another evidence of the role of SIRT3 in AMPK activation is its involvement in the increase of cytosolic calcium level that, in turn, activates CaMKKβ and promotes AMPK activity. It is thus worth hypothesizing that the AMPK/SIRT3 axis plays a vital role in renal lipotoxicity. 

Rodents and humans demonstrate the critical role of SIRT3 in metabolic diseases, highlighting the decrease in SIRT3 activity in several tissues such as the liver, the adipose tissue and the muscle [174,175]. Mice deficient for SIRT3 on an HFD also reveal accelerated hallmarks of MetS [176]. SIRT3 restoration is also beneficial in models of acute kidney injury (AKI) [177,178,179]. Indeed, maintaining SIRT3 activity was shown to prevent acute damage in renal tubular cells by maintaining the mitochondrial functional integrity and dynamics by normalizing mitochondrial protein acetylation [177,179]. Morigi et al. reported that in mice with cisplatin-induced AKI, tubular cell mitochondrial abnormalities were associated with decreased renal SIRT3 mRNA and protein expression, whereas treatment with AICAR, an activator of AMPK, improved renal function through the restoration of SIRT3 expression and activity. Mitochondrial protein acetylation normalized concomitantly with the upregulation of proximal tubular SIRT3 expression after AICAR treatment providing evidence of the role of AMPK activity in restoring SIRT3 deacetylase activity. These recent data shed light on the AMPK/SIRT3 pathway to maintain cellular homeostasis by improving mitochondrial functional integrity.

## 10. Conclusions and Future Directions

**Figure 5:**
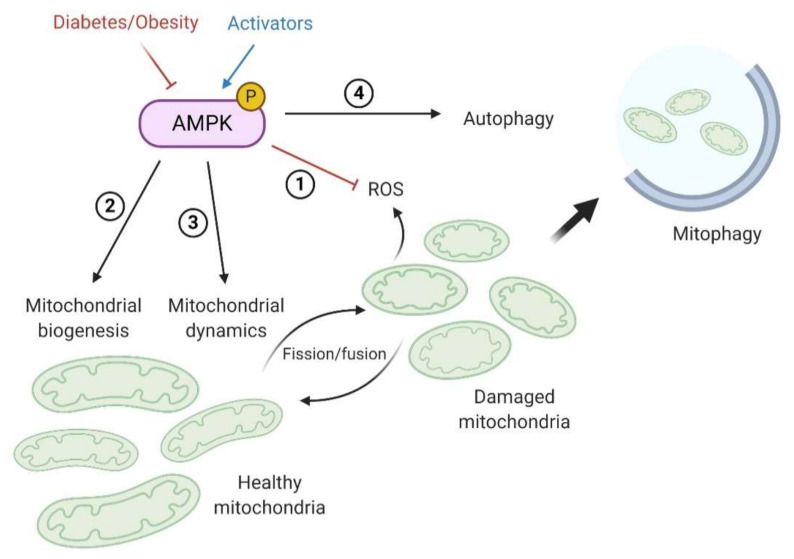
Mechanisms by which AMPK regulates mitochondrial homeostasis in the renal cell. Activation of AMPK in the context of obesity and diabetes leads to improved mitochondrial dysfunction and cellular metabolism by (1) decreasing oxidative stress (ROS) by inducing antioxidative mechanisms; (2) favoring mitochondrial biogenesis to increase mitochondrial density; (3) participating in the dynamic regulation of mitochondrial fission and fusion to facilitate their degradation by mitophagy; and (4) ultimately regulating mitochondrial quality control by the initiation of autophagy and mitophagy.

The downregulation of AMPK activity in obesity- and diabetes-induced CKD has been extensively reported in patients and in vivo and in vitro experimental models. Inhibition of AMPK activity in the kidney results from systemic metabolic dysregulation, signaling factors mediated by other dysfunctional tissues and organs (organ crosstalk), and intrarenal disturbances. Delineating the pleiotropic pathways regulated by AMPK and the potential impact of their dysregulation on renal cell function and metabolism remains a challenge. A better understanding of the AMPK pathway and the consequences of its dysregulation in metabolism-induced kidney diseases is thus essential to improve therapeutic strategies. Here, we present a more comprehensive and exhaustive review of the experimental and clinical evidence supporting the central role of AMPK in the development and progression of diabetes- and obesity-induced CKD. In particular, we summarized relevant data regarding the altered renal AMPK-mediated molecular mechanisms and critical targets in the context of excessive energy supply. In the kidney, AMPK is essential to maintain ATP levels to meet the high metabolic demands in renal cells to regulate renal transport. Moreover, there is a large body of evidence demonstrating that activating AMPK leads to the prevention of renal lipotoxicity and lipid accumulation by reducing FA synthase. In podocytes, glucose metabolism and associated insulin resistance are both linked with AMPK regulation. Beyond its role in glucose and lipid metabolism and as an energy sensor in the kidney, AMPK alleviates mitochondrial damage by at least four mechanisms (Figure 5): (1) AMPK is strongly implicated in the regulation and the response to ROS production by altered mitochondria, thus preventing damage induced by mtROS; (2) activation of AMPK favors mitochondrial biogenesis to enhance metabolism by inducing AMPK/SIRT1/PGC1-α pathway; (3) AMPK regulates mitochondrial dynamics and quality control, including mitochondrial fission/fusion; and (4) AMPK activity initiates autophagy flux and selectively degrades unhealthy mitochondria. Therefore, inhibition of renal AMPK is associated with poor outcomes and leads to lipotoxicity, insulin resistance, inflammation, fibrosis, and loss of renal function. Therefore, activating AMPK in metabolic disease and the associated renal injury represents a potentially critical therapeutic target [180]. 

Dynamic research has extended classical knowledge regarding AMPK beyond energy-sensing towards novel AMPK- related pathways and mechanisms in different models and pathologies. While in vitro studies of renal cells in the context of excess energy supply help investigate cellular and molecular mechanisms, they offer a reductive view of the complex regulation of in vivo AMPK that relies on several factors. However, in animal models of metabolic syndrome, diabetes, and obesity, AMPK activators demonstrate several unintended off-target effects. It is impossible to delineate the exclusive effects of these drugs on the kidney or specific renal cells. Therefore, more studies in models with tissue or cell type-specific genetic deletion or overexpression of AMPK in the context of metabolism-related kidney disease are required to delineate the pleiotropic roles of AMPK presented in this review.
